# A conceptual roadmap towards precision emergency surgery in adhesional small bowel obstruction

**DOI:** 10.1093/bjs/znag085

**Published:** 2026-07-29

**Authors:** Matthew J Lee

**Affiliations:** Department of Applied Health Sciences, University of Birmingham, Birmingham, UK; Department of Trauma and Emergency General Surgery, University Hospitals Birmingham, Birmingham, UK

## The problem

Adhesional small bowel obstruction (aSBO) is a major cause of morbidity and death, with a global incidence of 5.6 million per year, associated with ∼33 000 deaths, a loss of 1.86 million disability adjusted life years per year^1^, and incurs costs of $2300–$12 350 (approximately €2014–€10 816) for each admission^2^. Despite the importance and frequency of this condition, relatively little high-level evidence has been generated, with relatively few randomized controlled trials addressing stratification or treatment options. This paucity of evidence impedes our ability to deliver care that is stratified or personalized in a way that other surgical specialties can deliver.

## Current paradigms in aSBO care

Current treatment pathways focus on the binary decision of operation *versus* continued non-operative management. Early surgery might overtreat those who do not require an operation, running the risk of unnecessary morbidity, and increasing the risk of future aSBO due to new adhesions. Delayed surgery might lead to increased rates of intestinal ischaemia with consequent bowel resection, and its associated increased risk of death^3,4^. Treatment decisions are made with the goal of achieving resolution of gastrointestinal function, at minimal risk of morbidity, and minimization of mortality rate.

In modern practice, a patient with a history, symptoms, and examination compatible with aSBO will undergo diagnostic CT imaging at the point of admission. Decision-making is largely based upon this first assessment, supported by serial examination, and possibly the use of adjuncts such as water-soluble contrast^4,5^. If the decision is made for initial non-operative management, then this course is typically followed until surgery is mandated through failure to resolve or onset of ischaemia, resolution of SBO, or death. Consequently, current pathways detect deterioration rather than predict it.

Although we consider the described practice as ‘gold standard’, why might this not meet the standards we need for our patients? The answer is it is driven by our current diagnostic tools. CT scan, although highly sensitive and specific for the diagnosis of aSBO, assesses anatomy rather than function. Subclinical ischaemia may not be detected on this scan. Some scores have been proposed to help predict risk of ischaemia, but these have undergone limited validation^6,7^. At present, the only biomarker widely used in aSBO is lactate. This is a non-specific marker of ischaemia. A raised lactate may occur due to hypovolaemia associated with third-space losses, and a normal lactate might occur due to impaired organ flow and metabolite washout, giving incorrect measures in both settings. With this poor diagnostic performance, lactate has a negative predictive value of 96.3% and a positive predictive value of 43.6%^8^.

Although increasingly used, the therapeutic and prognostic value of water-soluble contrast agents has been questioned^5,9^. This change in perception may reflect evolving diagnostic pathways in aSBO. Earlier studies frequently used plain abdominal radiography to establish diagnosis, whereas contemporary pathways rely predominantly on CT imaging. Previous Cochrane reviews suggested that the presence of water-soluble contrast in the colon by 24 h post-administration was associated with a sensitivity of 97% and specificity of 96% for non-operative management^10^. This was largely based on trials using plain abdominal radiography as inclusion criteria^9^. Real-world data from the SBO-ACTION cohort study, which used CT-based diagnosis, found corresponding sensitivity and specificity values of 66% and 56% respectively^5^.

The central limitation of current aSBO care is that management decisions are based predominantly on static baseline assessment, despite aSBO representing a dynamic physiological process. Patients transition through evolving trajectories of obstruction, intestinal dysfunction, inflammatory activation, and tissue injury, yet contemporary pathways lack tools capable of repeated phenotyping of these changes. Consequently, clinical deterioration is often recognized only after overt decompensation has occurred, limiting opportunities for proactive intervention.

## aSBO as dynamic intestinal failure

However, this framework fails to address the central challenge in aSBO. We are not managing a single defined entity with localised effects and impacts; rather, aSBO should be conceptualized as acute intestinal failure with systemic physiological consequences. One should not dismiss this as a minor organ failure, as we see a 5% mortality rate linked to trials of non-operative management^5^. The impact of a non-functional gastrointestinal (GI) tract is not limited to the gut. aSBO brings with it third-space losses and volume depletion, meaning that 20% of patients will have an acute kidney injury (AKI) at the time of admission^4^. The body is also pushed into a catabolic state, which can lead to consumption of glycogen stores and ultimately skeletal muscle loss^11^, impairing function. This may also impede brain metabolism, leading to impaired cognition^12^. These sequelae are all easily measured; urea and electrolyte plus urine output measurement for renal function, sarcopenia on CT for catabolism, and cognitive state tests for delirium are all readily available. Aside from measures of vomiting and passing of stool or flatus, we lack reliable and reproducible measures of GI function^13^.

We also recognize that not all adhesions are the same; aSBO represents a heterogeneous group of biological and mechanical phenotypes rather than a single disease entity. Patients may differ not only in anatomical configuration of obstruction, but also in inflammatory response, physiological reserve, nutritional status, and ability to tolerate ongoing intestinal dysfunction. Consequently, identical radiological appearances may result in markedly different clinical trajectories. This parallels observations in trauma, where host response to injury coded by genetics can be as important as the injury^14^. Recognition of this heterogeneity is central to the development of precision emergency surgery, where treatment strategies are tailored not simply to the presence of obstruction, but to predicted disease behaviour and patient-specific risk. Some episodes of aSBO may resolve spontaneously due to transient kinking or reversible obstruction dynamics. Others may require surgery, due to dense fibrotic bands impeding flow of gastrointestinal content and compromising the vasculature. Of these domains, we only assess for intestinal ischaemia at baseline using a CT scan, and then with monitoring for peritonism. There is no routinely used assessment for anatomical configuration of obstruction, nor is generalized organ dysfunction proactively measured. Consequently, we must ask how we can make the transition from reactive to proactive care.

## From reactive to proactive care

This also demands that we should reconsider our treatment decision tree. Currently, decisions are made on the need for immediate surgery based upon the presence of ischaemia. Then, the decision is re-evaluated in a time-bound manner following a period of supportive care of around 72 h^3^. To move from this ‘failure to improve’ approach, we should consider the wider physiological impact of the condition. Patients who present with two organ dysfunction, for example aSBO and AKI, may be best served by aggressive surgical intervention. In parallel medical settings, we would not permit an organ failure causing downstream impact to go untreated, so why should this paradigm differ in aSBO? Reactive escalation following physiological deterioration is increasingly uncommon in other acute care pathways. In sepsis^15^, acute coronary syndromes^16^, and stroke^17^, treatment paradigms have shifted towards earlier identification of patients at risk of irreversible injury, with intervention delivered before overt organ failure develops. In contrast, aSBO pathways frequently tolerate prolonged periods of ongoing intestinal dysfunction provided frank ischaemia or peritonitis are absent^4^. This may represent a fundamental mismatch between the dynamic physiology of aSBO and the static frameworks currently used to guide management. Earlier identification of patients with progressive physiological stress may permit proactive intervention before irreversible intestinal or systemic injury develops.

## Domains of dynamic assessment

Multiple opportunities exist for the dynamic reassessment of aSBO to support risk stratification and personalization of care. This requires consideration of multiple domains; mechanical phenotype, GI functional phenotype, assessment for tissue injury, systemic physiology, and patient-reported recovery (*Table 1*).

**Table 1:** Summary of domains where dynamic reassessment to enable precision management might be viable

Dynamic reassessment domain	Current assessment methods	Current limitations	Emerging technologies/approaches	Potential clinical role
Mechanical phenotype	CT transition point, bowel calibre, closed-loop signs	Static snapshot with limited functional information	AI radiomics, geometric modelling, serial imaging, bowel acoustic sensing	Predict failure of NOM and identify high-risk obstruction configurations
Gastrointestinal functional phenotype	Vomiting, stool passage, flatus	Crude, subjective and intermittent measures	PROMs, digital symptom tracking, enteral tolerance monitoring, bowel acoustics	Identify recovery and non-recovery trajectories
Tissue injury/ischaemia	Lactate, CT ischaemic signs	Poor sensitivity and specificity, often late findings	iFABP, IL-6, endothelial biomarkers, metabolomics, photonics	Detect subclinical intestinal injury
Systemic physiology	NEWS score, urine output, AKI assessment	Non-specific downstream manifestations	Wearables, continuous physiology monitoring, haemodynamic profiling	Dynamic physiological phenotyping and early deterioration detection
Patient-reported recovery	Minimal routine assessment	Functional recovery poorly characterized	PRO-digi, QoL instruments, digital PROM platforms, wearables	Patient-centred recovery assessment

AI, artificial intelligence; AKI, acute kidney injury; iFABP, intestinal fatty acid–binding protein; IL-6, interleukin-6; NEWS, National Early Warning Score; NOM, non-operative management; PRO-digi, Patient-Reported Outcome Measure for Digestive Recovery; PROMs, patient-reported outcome measures; QoL, quality of life.

At present, mechanical phenotyping is limited to presence of aSBO, presence of internal hernia, and occasionally level of obstruction. It is conceivable that anatomical factors such as angulation might also be measured on a CT. This may reflect scope for AI-based tools to support prognostication^18^. More recently, bowel acoustic monitors have been explored to measure GI recovery^19^. It is plausible that such tools might be useful in measuring the strength and frequency of peristaltic waves attempting to clear a blockage, linked to the common symptom of colic.

Functional phenotypes, including degree of nausea/vomiting and pain, might also provide insight into how we predict who will settle without surgery. Intermittent clinician assessment may underestimate symptom burden and disease trajectory. Regular measurement of symptoms using patient-reported outcome measures (PROMs) and digital trackers might aid incorporation of this into prognostication. Such approaches have been deployed in oncological settings to monitor patient function^20^.

Several bodies of work exist in the detection of bowel ischaemia in acute mesenteric ischaemia, with few studies focused on aSBO. Given the poor performance of lactate, exploration of alternate biomarkers such as intestinal fatty acid–binding protein, interleukin-6, and D-lactate may have merit^21^. It may be that we rely on a combination of markers, or more on metabolic signatures of ischaemia. Photonics, a non-invasive measurement of cutaneous distribution of orally ingested fluorescein, has also shown promise in detection of gut membrane dysfunction^22^. This may represent a future opportunity for ‘bowel vital signs’, measured at the same time as pulse oximetry.

Systemic physiological phenotypes are currently inferred from intermittent clinical observations and downstream manifestations of intestinal dysfunction, including AKI, tachycardia, and biochemical evidence of hypovolaemia. These measures are non-specific and often detect deterioration only after overt physiological decompensation has occurred. Emerging technologies such as wearable physiological monitors^23^, continuous haemodynamic reassessment, and bioimpedance analysis^24^, may allow dynamic reassessment of fluid redistribution, tissue hydration and physiological reserve during aSBO. Such approaches may help identify patients failing to tolerate ongoing intestinal dysfunction before irreversible organ injury develops.

Patient-reported recovery phenotypes are rarely incorporated into contemporary aSBO pathways, with recovery typically defined by crude clinical markers such as tolerance of oral intake or passage of stool and flatus^13^. Such measures fail to capture the broader patient experience of gastrointestinal dysfunction, including nausea, appetite, pain, fatigue and confidence with eating^25,26^. Repeated assessment using GI recovery PROMs may allow identification of distinct recovery trajectories during and after admission. These approaches may improve identification of patients failing to recover following non-operative management and support more patient-centred treatment decision-making.

## Future precision pathways

**Figure 1:**
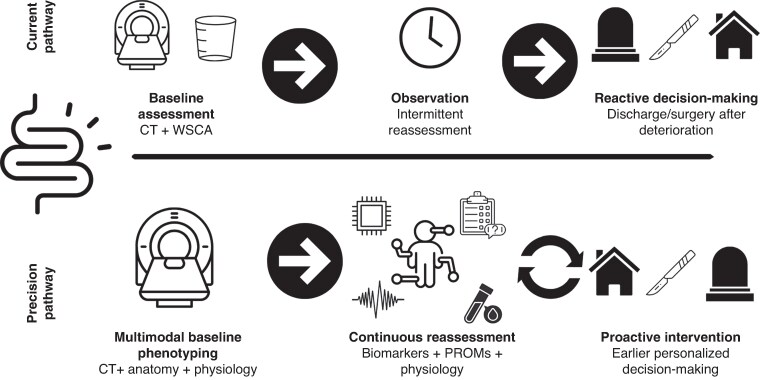
Contrast of current and precision SBO management pathways PROM, patient-reported outcome measure; WSCA, water-soluble contrast agent.

Future pathways are unlikely to rely upon a single biomarker or monitoring modality. Instead, precision management of aSBO may require integration of multimodal data streams including radiological phenotype, physiological monitoring, biomarkers, GI recovery measures, and patient-reported outcomes through dynamic longitudinal reassessment (*Fig. 1*). Such approaches may support continuously updated risk profiling capable of identifying changing trajectories of disease severity and treatment response. Advances in machine learning and digital health infrastructure may ultimately permit development of adaptive decision-support systems capable of guiding personalized treatment strategies in real time.

Successful implementation of dynamic reassessment pathways will require integration into existing emergency surgical workflows without increasing cognitive burden on clinicians. Future systems should therefore prioritize automated data capture, interoperability with electronic health records, and generation of clinically interpretable decision-support outputs. As with other digitally enabled pathways, careful evaluation will be required to ensure equitable access and avoidance of algorithmic bias.

It is probable that a new model of care would draw on data from across the highlighted domains to identify those with high baseline risk of ischaemia, or high levels of physiological stress manifested as multiorgan dysfunction. Iterative assessment of patients using clinical assessments, markers of GI perfusion and function, additional organ dysfunction, and patient function/recovery might provide longitudinal data to support personalized management strategies. Dynamic phenotyping may also support future adaptive trial designs in aSBO, where interventions are allocated or escalated according to evolving physiological trajectories rather than fixed baseline assessment alone. Such approaches may allow more efficient evaluation of personalized treatment strategies within heterogeneous emergency surgical populations.

There is a significant healthcare cost associated with aSBO, as well as a significant morbidity burden for our patients. It is time to advance the field, embrace new technologies, and strive to prevent harm from aSBO, rather than managing complications of the disease.

## Data Availability

This is a review. All relevant data cited.
